# The Influence of Short‐Term, Severe Low Energy Availability With Varying Protein Content on Substrate Metabolism, High‐Intensity Exercise Performance and Subjective Responses in Young Adults

**DOI:** 10.1002/ejsc.70125

**Published:** 2026-04-16

**Authors:** Bruna Aguera Reina, Rafaela Silverio Pinto, Gabriel Perri Esteves, João Vitor Mariano, Paul Swinton, Eimear Dolan

**Affiliations:** ^1^ Faculdade de Medicina FMUSP Applied Physiology and Nutrition Research Group—School of Physical Education and Sport and Center of Lifestyle Medicine Universidade de São Paulo São Paulo Brazil; ^2^ School of Health and Human Performance Robert Gordon University Aberdeen UK; ^3^ School of Arts Sciences and Humanities University of Sao Paulo Sao Paulo Brazil

**Keywords:** carbohydrate, fuelling, protein, relative energy deficiency in sports (REDs), sports nutrition

## Abstract

Low energy availability (LEA) may impact multiple biological processes, but the extent to which it affects exercise performance, and whether macronutrient composition modulates this, remains unclear. This randomised crossover trial investigated the influence of three dietary conditions with varying energy and protein availabilities on substrate metabolism, high‐intensity exercise performance and subjective responses in healthy active females (9) and males (10). Each condition lasted 5 days. On Day 1, resting metabolic rate, substrate metabolism during a 45‐min fixed‐load cycling test at 65% of the peak power output achieved during an aerobic capacity test, and Wingate performance were assessed. Participants were then provided with all foods to be consumed over the next 4 days, with the following caloric and protein content: adequate energy availability (AEA: 45 kcal·kgFFM^−1^ day^−1^; 1.5 g kg^−1^ protein); low energy availability (LEA: 15 kcal kgFFM^−1^ day^−1^, with all macronutrients reduced proportionally); and another LEA condition but with protein matched to AEA (LEA‐P). They returned to the laboratory on the 5th day to repeat all experimental tests. Participants also completed brief, semi‐structured interviews to explore their subjective responses to each condition. Results indicated that both the LEA and LEA‐P diets induced a shift towards increased fat oxidation at rest, but not during exercise, compared to AEA. In contrast, Wingate performance declined only in the LEA‐P trial compared to AEA, potentially due to its reduced carbohydrate content. Participants generally reported negative experiences during both calorie‐restricted trials, including hunger, fatigue, weakness and frustration, with symptoms appearing more pronounced in males and during the LEA trial.

## Highlights

- We conducted a randomised, controlled, crossover trial to examine physical and subjective exercise responses to three 5‐day diets differing in energy availability and protein content.
- Resting, but not exercising, substrate metabolism shifted towards increased fat and reduced CHO oxidation in both of the calorie‐restricted trials, with no additional effect of macronutrient composition.
- High‐intensity (Wingate) performance declined in the calorie‐restricted trial with higher protein/lower carbohydrate, but not when all macronutrients were reduced proportionally.
- Participants generally reported negative experiences during both calorie‐restricted trials, including hunger, fatigue, weakness and frustration, with symptoms appearing more pronounced in males.

## 1 Introduction

Ingesting sufficient energy to support all biological processes in addition to meeting the demands of exercise training can be challenging for athletes, particularly those who participate in weight‐sensitive or energetically demanding sports. Low energy availability (LEA) has been defined as a mismatch between dietary energy intake and exercise energy expenditure that leaves total energy needs unmet (Mountjoy et al. 2023), and its consequences can be neutral, positive or negative depending on many factors, including the duration and severity of exposure, nutrient availability and health and training status (Burke et al. 2023). More severe or prolonged LEA can, however, negatively impact many aspects of health and performance, as described in the Relative Energy Deficiency in Sport (REDs) model (Mountjoy et al. 2023).

Impaired energy metabolism and performance decrements are described as core parameters of the REDs model (Mountjoy et al. 2023), which is of particular concern to athletes, considering that optimising performance is generally their primary goal. However, the evidence base on how LEA impacts these parameters is both limited and, at times, inconsistent (Areta 2023; Melin et al. 2024; Jeppesen et al. 2025). Most research to date is observational and, given the difficulty of accurately measuring energy availability in free‐living situations (Burke et al. 2018), generally relies on indirect estimates of LEA, such as questionnaires, comparison of estimated with predicted resting metabolic rate (RMR) or reproductive status, rendering it difficult to determine causality (Jeukendrup et al. 2024). Controlled trials remain scarce (Jeppesen et al. 2025), and those available generally report mixed results on performance outcomes such as time‐to‐exhaustion tests or time trials, countermovement jumps and Wingate tests (Smith et al. 2025; Kojima et al. 2020; Oxfeldt et al. 2024; Jurov et al. 2022).

The influence of LEA on exercise performance likely depends on the nature of the exercise task, as well as the duration and severity of LEA exposure. A potential limitation of many shorter‐term experimental studies is that they often induce LEA through a combination of increased exercise energy expenditure and reduced calorie intake. Although this may be representative of longer‐term real‐world scenarios, the added stress of unaccustomed exercise in a short‐term laboratory setting may confound attempts to isolate the effect of LEA per se. Furthermore, it is challenging to distinguish effects of LEA from those caused by inadequacy of important nutrients, such as carbohydrate and protein. Increasing, or at least maintaining, protein intake is often recommended during periods of energy restriction (Hector and Phillips 2018; Witard et al. 2025), the intention being to protect against lean mass loss and to promote recovery and training adaptations. Macronutrients are, however, compositional variables of total energy intake, and altering one macronutrient (e.g., protein) while maintaining total intake constant will invariably influence the others. For example, maintaining or even increasing protein intake during periods of LEA necessitates a greater reduction of carbohydrates and fats, which are also fundamental to supporting health and performance. Disentangling these parameters is challenging but necessary to design nutritional programmes optimised to support both health and performance. With this in mind, the aim of this study was to investigate the influence of three experimental diets that varied in their energy and macronutrient availability on parameters related to exercise performance in a group of healthy young adults.

## 2 Methods

### 2.1 Research Design

This study used a randomised, crossover, experimental design. All participants presented to the laboratory on 8 occasions. The first two visits were used to collect preliminary data and for familiarisation, with the experimental trials conducted across the remaining 6 visits. During the first session, participants underwent aerobic capacity testing, which was used to determine the load for subsequent experimental tests. Preliminary data, including resting metabolic rate (RMR), skinfolds, height and weight, were also collected. Instructions for maintaining a 3‐day food diary were provided, and participants were requested to complete this prior to the start of experimental testing. In the second session, participants were familiarised with the experimental exercise test, which comprised a steady‐state cycling protocol conducted at 65% of the peak power output achieved within the aerobic capacity test conducted in Session 1, followed by a Wingate test. They then took part in three experimental trials in a randomised order, separated by a minimum of 9 days and with different experimental diets provided in each one, namely: (1) adequate energy availability (AEA); (2) low energy availability with all macronutrients reduced proportionally (LEA); and (3) low energy availability with the same energy content as LEA, but with protein intake matched to AEA (LEA‐P). Experimental test sessions, comprising RMR assessment and cycling tests (fixed‐load and Wingate), took place on the first and last day of each 5‐day protocol. The outcomes of interest for this study were energy expenditure and substrate use at rest and during the steady‐state exercise test, Wingate performance and perceived exertion throughout the exercise protocols. Participants also underwent a brief, semi‐structured interview at the end of each condition, where they described their subjective experience of each diet, along with its perceived impact on exercise performance. These outcomes were secondary measures collected during a larger study, the aim of which was to investigate the influence of these diets on the bone biomarker and inflammatory cytokine response to exercise.

### 2.2 Participants

Healthy females and males aged 18–45 years were recruited. They were active but not highly trained, defined as participating in at least 150 min of moderate activities but less than 10 h of dedicated training in a specific sport per week. The decision to recruit active nonathletes was because elite athletes have modality‐specific dietary and training requirements, which may influence their response to stressors such as LEA. Given the current lack of controlled experimental data on these topics, we deemed it important to first focus on general responses and to use these results to generate hypotheses based on more specific populations such as elite athletes in different modalities. Exclusion criteria included the following: (1) currently following an energy‐ or nutrient‐restricted diet; (2) having substantial weight fluctuations in the last 6 months; (3) having any injury or medical condition that could affect participation in the study. The sample size was determined based on the primary outcome from the overarching study, namely, the bone biomarkers CTX‐1 and P1NP, which indicated that ∼20 individuals (10 females and 10 males) would be adequate to detect estimated effects. As the outcomes presented herein were secondary outcomes from a larger study, they were considered exploratory, and so power calculations were not performed. This study was approved by the local ethics and research committee (CAAE 33784720.7.0000.0068), and all participants provided written informed consent prior to participation.

### 2.3 Dietary Interventions

The energy content of the three experimental diets was expressed relative to fat‐free mass (FFM) and equalled 45, 15 and 15 kcal kgFFM^−1^·day^−1^ for AEA, LEA and LEA‐P, respectively. FFM was estimated based on the sum of seven skinfolds assessed using ISAK criteria and converted to body fat percent using the Jackson–Pollock equation. Participants were instructed to maintain their usual daily activities but not to undertake any structured exercise training during the experimental period, so no adjustment was made for exercise energy expenditure. The macronutrient composition of the AEA trial was approximately 60% carbohydrates, 15% protein and 25% fat, with the protein contribution maintained at 1.5 g kg^−1^ day^−1^. Within the LEA trial, all macronutrients were reduced proportionally, maintaining similar percent contributions as in AEA, whereas in LEA‐P, the protein content was maintained at 1.5 g kg^−1^ day^−1^, which required a larger reduction in carbohydrate and fat contribution to meet the target of 15 kcal kgFFM^−1^ day^−1^. An overview of the energy and macronutrient composition of each dietary condition is presented in Supporting Information S1. All experimental diets and their nutritional compositions were calculated using the WebDiet software, and all foods were packaged and delivered to each participant at the start of each experimental trial. The experimental diet order was randomly allocated using a 3 × 3 Latin square design.

### 2.4 Experimental Protocols

Participants arrived in the laboratory between 07:00 and 09:00 after an overnight fast. They were instructed to maintain their usual dietary habits and to avoid any unusual or strenuous exercise for at least 48 h prior to the test. Testing for female participants was not standardised to a specific menstrual cycle phase, as pretesting and post‐testing within each dietary condition occurred within a 5‐day period, a duration unlikely to be associated with substantial changes in hormonal status. RMR was assessed using indirect calorimetry (K5, COSMED). Participants were then provided a standardised breakfast and, 30 minutes later, underwent a 45‐min fixed‐load test conducted at 65% of the peak power output achieved during the aerobic capacity test conducted during the preliminary sessions. The aerobic capacity test started at 100 W for males and 50 W for females, and the load was increased by 25 W every 3 min until volitional fatigue. Expired gases and ventilation were continuously assessed throughout both the incremental and fixed‐load tests using indirect calorimetry (K5, COSMED). Participants self‐rated their perceived exertion at the end of each stage using a 6–20‐point Borg scale. On completion of the fixed‐load test, participants had a 5‐min recovery period and then completed a 30‐s Wingate test.

### 2.5 Subjective Response to the Dietary Conditions and Qualitative Analysis

At the end of each experimental period, participants underwent a brief, semi‐structured interview about their experience of each diet. Responses were assessed using a thematic approach. Initially, all responses were transcribed and organised by experimental diet. A fluctuant reading was then conducted to familiarise with the material and to identify key themes. Individual phrases were then organised within these themes using a ‘cut and sort’ approach. The analysis was conducted in duplicate, whereby two researchers initially agreed on the broad themes and then independently cut and sorted the data before meeting to compare analyses, resolve disagreements and agree on findings of interest via discussion.

### 2.6 Statistical Analysis

Continuous variables are presented as means and standard deviations, whereas categorical variables are reported as counts and proportions. Differences between sexes were compared using Welch’s *t*‐test. Linear mixed models were used to investigate the influence of each experimental condition on outcomes of interest (body mass and sum of 7 skinfolds; resting and exercise energy expenditure and substrate use; RPE throughout the fixed‐load test; total work done and mean and peak power during the Wingate test). Dietary condition was included as a fixed effect and participants as a random intercept. Differences in baseline values were included as covariates (Mehrota 2014), and any missing baseline data were imputed using the mean of all available pretest measurements. Missing postintervention values were not imputed. The model analysing RPE also included a fixed effect of time (namely, time throughout the exercise test) and an interaction between diet and time. The main model included all participants, both male and female. Exploratory models to assess whether sex influenced the response to the dietary interventions were also conducted by testing for interactions between dietary condition and sex for all outcomes. Type III ANOVA tables were used to determine the statistical significance of model terms, and when significant, baseline‐adjusted contrasts between diets (i.e., AEA vs. LEA, AEA vs. LEA‐P and LEA‐P vs. LEA) were computed with Tukey's adjustment and reported as mean differences and their 95% confidence intervals. All analyses assumed an alpha of 5% and were executed in R using ‘lmerTest’ for fitting mixed models and ‘marginaleffects’ for calculating comparisons and model predictions.

## 3 Results

Participant characteristics are described in Table 1. Nineteen participants took part in the study (9 females and 10 males). Of these, 16 completed all tests, and 3 missed one experimental trial (1 AEA and 2 LEA‐P). On average, males were taller, had lower body fat percent, higher FFM and wider waist circumference than females, with all other outcomes being similar between the sexes.

**TABLE 1:** Participant characteristics.

Characteristics	Total (*n* = 19)	Females (*n* = 9)	Males (*n* = 10)	*p*‐value
Age (yrs)	29 ± 8	31 ± 7	27 ± 8	0.30
Height (cm)	171 ± 8	166 ± 8^a^	174 ± 7^a^	0.03
Weight (kg)	72 ± 11	69 ± 11	75 ± 10	0.20
BMI (weight·height^−2^)	24.78 ± 2.71	24.86 ± 3.05	24.71 ± 2.54	0.90
Body fat (%)	17.9 ± 5.7	21.9 ± 4.7^a^	14.7 ± 4.4^a^	0.005
Fat‐free mass (kg)	59 ± 9	53 ± 5^a^	64 ± 8^a^	0.003
VO^2^_Peak_ (ml·kg·min^−1^)	43 ± 11	38 ± 10	48 ± 11	0.07
Waist circumference (cm)	77.1 ± 6.1	73.4 ± 5.5^a^	80.4 ± 4.6^a^	0.009
Hip circumference (cm)	101 ± 9	105 ± 10	98 ± 6	0.07
Abdominal circumference (cm)	80.3 ± 5.6	79.4 ± 6.7	80.9 ± 4.9	0.60

*Note:* Data are presented as mean (standard deviation).

^a^ Indicates a significant difference between females and males.

### 3.1 Resting and Exercise Energy Expenditure, Substrate Use, Body Mass and Skinfolds

Data on resting energy expenditure and substrate use are reported in Table 2. Neither RMR nor RMR·FFM^−1^ was influenced by the experimental diets (*F* = 18.3, *p* = 0.06 and *F* = 2.35, *p* = 0.12, respectively). A main effect of diet (*F* = 4.47, *p* = 0.02) was obtained for resting respiratory exchange ratio (RER), with results showing lower values for the LEA‐P compared with the AEA diet (mean difference: −0.06 [95% CI: −0.12 to −0.01, *p* = 0.03]), no difference between the LEA and AEA diets (mean difference: −0.04 [95% CI: −0.09 to 0.09, *p* = 0.11]) and no difference between LEA‐P and LEA (mean difference: −0.02 [95% CI: −0.08 to 0.04, *p* = 0.63]). A main effect of diet was also observed for resting substrate use (*F* = 5.12, *p* = 0.01), with carbohydrate oxidation significantly reduced in both the LEA (mean difference: −16.1% [95% CI: −30.1% to −2.1%, *p* = 0.03]) and LEA‐P (mean difference: −21.9% [95% CI: −37.6% to −6.2%, *p* = 0.01]) conditions compared to AEA, but no difference between LEA‐P and LEA (mean difference: −5.8% [95% CI: −21.5% to 10.0%, *p* = 0.46]). In contrast, fat oxidation (*F* = 5.12, *p* = 0.01) was higher in both the LEA (mean difference: 16.1% [95% CI: 2.1%–30.1%, *p* = 0.03]) and LEA‐P (mean difference: 21.9% [95% CI: 6.2%–37.6%, *p* < 0.01]) conditions compared to AEA, with no difference between LEA‐P and LEA (mean difference: 5.8% [95% CI: −10.0% to 21.5%, *p* = 0.46]). Dietary condition did not influence any of these parameters during the exercise bout, nor body mass or the sum of 7 skinfolds. Exploratory models indicated no influence of sex on any of these findings (*p* > 0.05).

**TABLE 2:** Energy expenditure and substrate metabolism at rest and during exercise.

	AEA		LEA		LEA‐P	
	Pre	Post	Pre	Post	Pre	Post
Rest						
RMR (kcal)	1930 ± 806	1982 ± 533	1973 ± 407	1840 ± 465	2073 ± 769	2113 ± 529
RMR/FFM (kcal·kg^−1^)	34 ± 14	33 ± 8	34 ± 5	32 ± 5	34 ± 11	36 ± 7
CHO oxidation (%)	35 ± 19	42 ± 29	44 ± 26	32 ± 16*	40 ± 20	23 ± 15*
Fat oxidation (%)	65 ± 19	58 ± 29	56 ± 26	68 ± 16*	60 ± 20	77 ± 15*
RER	0.81 ± 0.06	0.83 ± 0.09	0.84 ± 0.08	0.80 ± 0.04	0.82 ± 0.06	0.77 ± 0.05*
Exercise						
Energy expenditure (kcal·min^−1^)	13.24 ± 3.15	12.93 ± 2.28	12.81 ± 3.33	12.52 ± 2.73	12.58 ± 3.01	12.82 ± 3.12
Fat oxidation (kcal·min^−1^)	6.3 ± 4.0	6.5 ± 3.3	5.7 ± 3.4	5.8 ± 3.7	6.1 ± 3.7	7.1 ± 3.7
CHO oxidation (kcal·min^−1^)	6.97 ± 2.87	6.46 ± 3.20	7.14 ± 2.75	6.76 ± 2.97	6.49 ± 2.74	5.76 ± 2.61
Fat oxidation (%)	45 ± 22	50 ± 24	43 ± 21	45 ± 25	46 ± 25	53 ± 24
CHO oxidation (%)	55 ± 22	50 ± 24	57 ± 21	55 ± 25	54 ± 25	47 ± 24
RER	0.87 ± 0.07	0.86 ± 0.07	0.87 ± 0.07	0.87 ± 0.08	0.87 ± 0.08	0.86 ± 0.07
Body composition						
Body mass (kg)	72 ± 10	71 ± 10	73 ± 10	71 ± 10	74 ± 9	72 ± 9
Sum of 7 skinfolds (mm)	98 ± 31	95 ± 28	101 ± 33	96 ± 29	98 ± 34	93 ± 31

*Note:* Data are presented as mean ± standard deviation.

Abbreviations: FFM, fat‐free mass; RER, respiratory exchange ratio; RMR, resting metabolic rate.

^*^ Indicates a statistically significant difference compared to AEA (*p* < 0.05).

### 3.2 Perceived Exertion Throughout the Fixed‐Load Test

**FIGURE 1:**
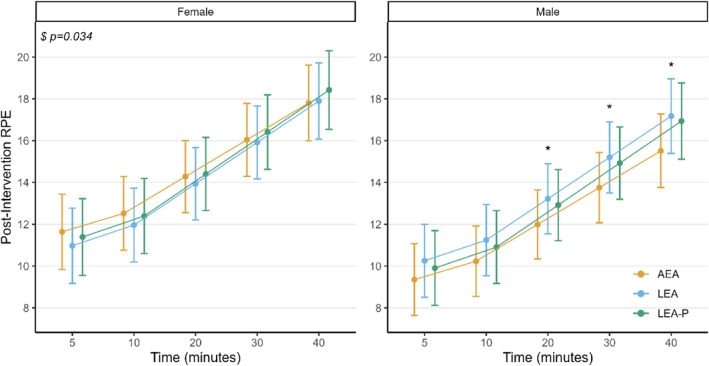
Rate of perceived exertion throughout the exercise tests aggregated by sex. $ denotes a significant diet–sex interaction (*p* = 0.034). * denotes a statistically significant difference between LEA and AEA at the specified time.

Perceived exertion throughout the exercise test increased across time (*F* = 388.1, *p* < 0.001) but was not influenced by diet (*F* = 0.59, *p* = 0.55) when considering all participants as a single group. The exploratory model indicated a significant interaction between sex and condition (*F* = 3.43, *p* = 0.034), whereby males reported higher RPE after the LEA condition compared to AEA at 20 (mean difference: 1.23 [95% CI: 0.11–2.35, *p* = 0.03]), 30 (mean difference: 1.45 [95% CI: 0.21–2.68, *p* = 0.02]) and 40 (mean difference: 1.66 [95% CI: 0.12–3.21, *p* = 0.03]) minutes into the exercise test (see Figure 1). No difference in RPE between dietary conditions was observed at any other time point for males, nor at any time point for females. No significant RPE differences were observed between LEA and LEA‐P (all *p* > 0.05).

#### 3.2.1 Performance in the Wingate Test

**FIGURE 2:**
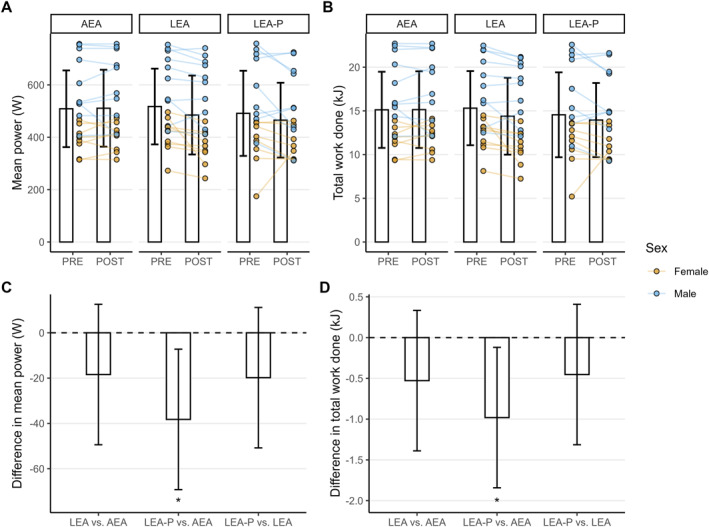
Effect of dietary condition on performance measurements. Panels A and B show the mean plus one standard deviation, alongside individual data coloured according to sex, for mean power and total work done. Panels C and D show pairwise contrasts between diets, alongside 95% confidence intervals. * indicates statistically significant differences (*p* < 0.05).

Dietary condition significantly influenced both total work done (*F* = 4.26, *p* = 0.02) and mean power (*F* = 4.98, *p* = 0.01) during the Wingate test (see Figure 2), with pairwise contrasts indicating reduced performance in LEA‐P compared to AEA (TWD mean difference: −0.98 [95% CI: −1.84 to −0.12, *p* = 0.024]; mean power mean difference: −38.2 [95% CI: −69.2 to −7.2 W, *p* = 0.015]). No difference was observed for these parameters between AEA and LEA or LEA‐P and LEA, nor were there any interactions between dietary condition and sex (*p* > 0.05).

### 3.3 Participant Perception of Each Dietary Condition

Findings from the postdiet interviews were organised in three themes: (1) hunger and satiety; (2) self‐reported physical and psychological state; and (3) perceived influence on test performance. Regarding the AEA diet, the majority of participants reported a high level of satiety, with some commenting on the volume of food and having difficulty consuming it all. There were minimal negative symptoms reported with the AEA diet, with just one female indicating some gassiness and one male indicating some nausea due to the amount of food. Considering overall energy levels and sense of well‐being with the AEA diet, the majority reported feeling similar to their usual state, with no major positive or negative effects, although two males commented that they may have felt less energetic than usual. In relation to their experience of the exercise test, the group as a whole felt that the diet had a neutral or slightly positive influence on their test performance.

In contrast, participants commonly reported high levels of hunger throughout the LEA diet. There was some contrast in how this manifested across time, with some reporting greater challenges in the first couple of days that then subsided, whereas others reported the opposite, saying that their hunger increased throughout the week. Participants' overall perception of their vitality and well‐being on the LEA diet was largely negative, with many commenting that they felt weak, tired, had headaches and felt irritable and stressed. As a group, they also reported a worsened experience during the exercise test, reporting feelings of heaviness in the legs and tiredness throughout. Participants' responses to the LEA‐P diet were generally similar to those reported for LEA, consistently reporting feelings of weakness, fatigue and headaches. Interestingly, their comments regarding hunger/satiety and their perceptions of the exercise test were more mixed, with some reporting that they felt less hungry and that they performed well compared to previous diets, whereas others reported the opposite. Overall, males appeared to have more difficulty in following both LEA diets, with negative comments generally appearing more frequently and strongly compared to females. A difficulty in interpreting these responses was that the participants tended to compare their diets with what they had undergone before. Given that the dietary condition order was randomised, this may have skewed their perceptions and influenced these results.

## 4 Discussion

The aim of this study was to investigate the influence of three experimental diets with varying energy and macronutrient compositions on aspects of exercise performance. The primary findings are as follows: (A) Both energy‐restricted diets induced a shift in substrate metabolism at rest, characterised by reduced carbohydrate oxidation and greater reliance on fat as a fuel source. This finding did not persist during exercise, with similar energy expenditure and substrate metabolism observed across all dietary conditions. (B) Anaerobic exercise performance was negatively influenced only during the LEA‐P condition, likely due to the reduced carbohydrate availability in that diet. (C) Participants generally had a negative response to both energy‐restricted diets, reporting feelings of hunger, weakness and a perceived negative impact on exercise capacity and performance. Males reported a higher RPE during the fixed‐load test following the LEA diet compared to AEA.

Metabolic flexibility, defined as the body's ability to adapt its metabolism based on energy availability and demand, is key to athletic performance. Low energy availability may induce compensatory alterations in energy metabolism to conserve energy during times of scarcity, for example, via reduced RMR or shifting metabolism to rely more on fat as opposed to carbohydrate oxidation. Within this study, RMR was not influenced by the calorie‐restricted dietary interventions, which is in agreement with other short‐term studies (Jurov et al. 2022; Nolte et al. 2025; Kuikman et al. 2025; Caldwell et al. 2024) and likely reflects the short exposure time to the different dietary conditions. In contrast, resting substrate metabolism was altered in both calorie‐restricted diets, indicating increased reliance on fat oxidation and reduced carbohydrate use. The human body has a well‐documented capacity to quickly and reversibly alter energy allocation and metabolism when energetically stressed (Shirley et al. 2022). This shift in substrate metabolism at rest likely represents an adaptive response to conserve carbohydrate availability, as both LEA diets had substantially less carbohydrate than the AEA condition. Indeed, there did appear to be a larger shift in substrate use in the LEA‐P trial, which also had a lower carbohydrate content; however, this finding was not statistically significant. Interestingly, the same shift in substrate use was not observed during exercise. Energy allocation strategies are primarily determined by necessity (Shirley et al. 2022), and it is possible that the stress imposed by the exercise bout encouraged prioritisation of available energy and carbohydrate to sustain it. It was also interesting to note that body mass did not significantly change in any of the three dietary conditions, although mean group data indicated an average reduction of approximately 2 kg in both LEA conditions. There was, however, also a nonsignificant average reduction of about 1 kg in the AEA trial, which may have obscured differences between conditions. None of these changes were statistically significant, likely due to intraindividual variability; however, it does seem that some participants lost weight, even while consuming a theoretically adequate energy intake. Under‐consumption of the prescribed AEA diet seems unlikely, as we contacted participants daily to verify adherence. It is possible, however, that improved dietary quality during the intervention, such as a greater reliance on un‐ or minimally processed foods and, in turn, reduced sodium and increased fibre, may have influenced body mass through hydration shifts or bowel movements. These parameters were not directly investigated, however, and as such, this hypothesis cannot be confirmed or ruled out within this study.

Despite similar alterations in substrate metabolism in both energy‐restricted conditions, performance in the Wingate test was negatively impacted only in LEA‐P. Previous research examining low energy availability over similar time periods (1–21 days) has reported mixed effects on this test, with null (Mettler et al. 2010; Mourier et al. 1997; Fogelholm et al. 1993; McMurray et al. 1991), negative (McMurray et al. 1991) and even positive (Fogelholm et al. 1993; Zachwieja et al. 2001) effects reported. These conflicting findings likely relate to differences in intervention duration and carbohydrate availability. Within this study, it seems plausible that the reduced Wingate performance observed in the LEA‐P condition was due to the lower CHO availability in this condition compared to both LEA and AEA, which may have important performance implications for athletes whose sports involve brief, high‐intensity bursts of effort. It is important to highlight, however, that no differences were observed between LEA and either AEA or LEA‐P, and we did not directly measure muscle glycogen content. As such, our suggestion that the reduced performance in LEA‐P but not LEA compared to AEA is attributable to carbohydrate availability is a hypothesis. Testing this would require an additional energy‐restricted condition in which carbohydrate rather than protein is maintained. Adding this condition was beyond the scope of this study, but this could be an interesting question to address in follow‐up studies. It is also important to consider that although performance was not significantly impacted in the LEA trial compared to AEA, protein intake in that trial was also very low (approximately 0.45 g·kg^−1^), which may have important performance, recovery and health implications if sustained across the longer term. The reality is that as macronutrients are compositional variables of total energy intake, it is not possible to maintain adequacy in all macronutrients when energy intake is severely restricted. As such, prioritising one necessarily leads to trade‐offs against others, which should be taken into consideration when determining energy availabilities and macronutrient ratios in different contexts.

Considering participants' self‐reported energy levels and performance, as a group, they struggled with both calorie‐restricted diets, reporting sensations of hunger, fatigue, weakness and headaches. These responses appeared to be more pronounced in the LEA compared to the LEA‐P diet, which may relate to the role of protein in promoting satiety (Raubenheimer and Simpson 2019). Interestingly, the male group generally had a worse subjective response to the calorie‐restricted diets, more consistently and strongly expressing their difficulty in following the diets and in coping with the lack of food. They also reported a higher rating of perceived exertion in the LEA trial compared to AEA—a finding which was not apparent in the female group. It is possible that this relates to the differing shape of the curves seen in RPE (see Figure 1), whereby the female group reported higher perceived exertion throughout the tests, ending all at close to maximal exertion. As such, the lack of observed difference here may reflect a ceiling effect, as described by one of the participants in the interviews: *‘I died in all the tests, but I died the most after the LEA diet’*. It is also plausible that males may be more sensitive to the effects of calorie restriction—at least in terms of their subjective experience—as their interview responses generally indicated a worse experience in the calorie‐restricted trials than was reported by females.

This study has limitations that are important to consider when interpreting results. Brief, controlled experiments, as described herein, are limited in representing the real‐life experience of active individuals with LEA. In particular, the negative influences of low energy availability are generally believed to occur after more prolonged exposures, and this brief intervention provides only a ‘snapshot’ of the short‐term impact of LEA with varying macronutrient compositions on exercise metabolism and performance. We opted to use controlled standardised exercise protocols to facilitate isolation of any changes to the dietary conditions themselves, as opposed to other factors such as altered exercise intensity or volume. This approach does, however, reduce the ecological validity of our findings, and future research should strive to investigate how energy availability and macronutrient composition influence tests that better reflect real‐life sporting outcomes. Furthermore, our population were healthy and active but were not athletes, and it is plausible that high‐performance athletes in different modalities may respond differently, representing another important avenue for ongoing research. The only between‐sex difference that we observed was for RPE, whereby males reported a higher perceived exertion in the LEA trial compared to AEA. It is important to consider, however, that the main effect of any intervention involving nutritional manipulation is likely to be far larger than the interaction between the intervention and sex (Dolan et al. 2024), rendering far larger sample sizes necessary to more conclusively determine whether sex differences are present or not.

## 5 Conclusion

This study examined the influence of a 5‐day intervention period involving diets with varying energy and macronutrient compositions on aspects of substrate metabolism, high‐intensity exercise performance and subjective responses in a group of healthy, active adults. Results indicated that both calorie‐restricted diets induced a shift in substrate metabolism favouring fat oxidation at rest, but no change during exercise. In contrast, high‐intensity exercise performance was negatively impacted only during the LEA‐P trial, likely due to the reduced carbohydrate content of this diet. Participants generally had a negative experience on both calorie‐restricted trials, reporting feelings of hunger, fatigue, weakness and frustration, with these feelings appearing most pronounced for males and within the LEA trial. Our results highlight the importance of maintaining adequate energy and carbohydrate availability, particularly during competitive periods. This is not to say that protein is not important and should not be considered, particularly in terms of protecting longer‐term musculoskeletal health. The reality is, however, that in situations of severe calorie restriction, it is difficult, if not impossible, to maintain adequacy of all macronutrients, and care should be taken to balance the benefits versus risks of undereating specific nutrients in specific contexts.

## Ethics Statement

Ethical approval to undertake the study was obtained from the local committee (CAAE 33784720.7.0000.0068), and all participants voluntarily signed informed consent forms prior to their participation.

## Conflicts of Interest

The authors declare no conflicts of interest.

## Supporting information


Supporting Information S1

## Data Availability

Data are freely available from the corresponding author upon request.
