# Supplementary material for: The Influence of Short‐Term, Severe Low Energy Availability With Varying Protein Content on Substrate Metabolism, High‐Intensity Exercise Performance and Subjective Responses in Young Adults
Source: Eur J Sport Sci. 2026 Apr 16;26(5):e70125. doi: 10.1002/ejsc.70125 (PMC13086566; doi:10.1002/ejsc.70125)
Supplement: Supplementary file 1 — Supporting Information S1 [file EJSC-26-e70125-s001.docx]

**The Influence of Short-Term, Severe Low Energy Availability with Varying Protein Content on Substrate Metabolism, Exercise Performance and Perceptual Responses in Young Adults.**

**SUPPLEMENTARY FILES**

**Table 1:** Experimental dietary composition

|  | **Women** | | | | **Men** | | | |
| --- | --- | --- | --- | --- | --- | --- | --- | --- |
|  | **Habitual** | **AEA** | **LEA** | **LEA-P** | **Habitual** | **AEA** | **LEA** | **LEA-P** |
| **Energy (Kcal)** | 1680 ± 309 | 2376 ± 229 | 799 ± 74 | 817 ± 54 | 2185 ± 337 | 2871 ± 378 | 957 ± 126 | 972 ± 124 |
| **Energy (Kcal·FFM⁻¹)** | 32 ± 7 | 45 ± 0 | 15 ± 0 | 15 ± 0 | 38 ± 8 | 45 ± 0 | 15 ± 0 | 15 ± 0 |
| **PAL (Kcal·RMR)** | - | 1.29 ± 0.28 | 0.45 ± 0.09 | 0.46 ± 0.10 | - | 1.47 ± 0.43 | 0.48 ± 0.07 | 0.46 ± 0.10 |
| **Protein (g)** | 128 ± 40 | 102 ± 15 | 30 ± 3* | 107 ± 13 | 124 ± 29 | 113 ± 16 | 36 ± 5 | 114 ± 16 |
| **Protein (g·kg⁻¹)** | 1.91 ± 0.70 | 1.5 ± 0 | 0.44 ± 0.03 | 1.5 ± 0 | 1.64 ± 0.34 | 1.5 ± 0 | 0.48 ± 0.03 | 1.5 ± 0 |
| **Protein (%)** | 31 ± 10 | 17 ± 1 | 15 ± 0 | 52 ± 4 | 23 ± 5 | 16 ± 1 | 15 ± 0 | 47 ± 3 |
| **Carbohydrate (g)** | 183 ± 51 | 354 ± 32 | 120 ± 11 | 63 ± 7 | 275 ± 52 | 428 ± 58 | 144 ± 19 | 90 ± 13 |
| **Carbohydrate (g·kg⁻¹)** | 2.74 ± 0.84 | 5.27 ± 0.42 | 1.74 ± 0.12 | 0.89 ± 0.16 | 3.78 ± 1.28 | 5.69 ± 0.42 | 1.91 ± 0.12 | 1.19 ± 0.14 |
| **Carbohydrate (%)** | 43 ± 9 | 60 ± 3 | 60 ± 0 | 31 ± 4 | 50 ± 6 | 60 ± 1 | 60 ± 0 | 37 ± 2 |
| **Fat (g)** | 46 ± 15 | 63 ± 11 | 22 ± 2 | 15 ± 2 | 69 ± 21 | 79 ± 10 | 27 ± 3 | 17 ± 2 |
| **Fat (g·kg⁻¹)** | 0.69 ± 0.26 | 0.93 ± 0.14 | 0.32 ± 0.02 | 0.22 ± 0.03 | 0.92 ± 0.26 | 1.05 ± 0.07 | 0.35 ± 0.02 | 0.23 ± 0.03 |
| **Fat (%)** | 32 ± 16 | 24 ± 3 | 25 ± 0 | 17 ± 2 | 36 ± 7 | 25 ± 0 | 25 ± 0 | 16 ± 1* |

Data are presented as mean ± standard deviation.

**Table 2:** Energy expenditure and substrate metabolism and rest during exercise aggregated by sex.

|  | **Women** | | | | | | | **Men** | | | | | |
| --- | --- | --- | --- | --- | --- | --- | --- | --- | --- | --- | --- | --- | --- |
|  | **AEA**  **(pre)** | **AEA**  **(post)** | **LEA**  **(pre)** | | **LEA**  **(post)** | **LEA-P**  **(pre)** | **LEA-P**  **(post)** | **AEA**  **(pre)** | **AEA**  **(post)** | **LEA**  **(pre)** | **LEA**  **(post)** | **LEA-P**  **(pre)** | **LEA-P**  **(post)** |
| ***Resting*** | | | | | | | | | | | | | |
| **RMR (kcal)** | 1706±702 | 1917±469 | | 1868±466 | 1810±340 | 1579±428 | 1857±373 | 2232±923 | 2076±550 | 2196±353 | 2020±514 | 2420±714 | 2203±479 |
| **RMR·FFM^-1^** | 33 ±1 | 36 ±6 | | 34 ±7 | 34 ±7 | 28 ±8 | 34 ±7 | 35 ±13 | 33 ±8 | 34 ±3 | 32 ±5 | 37 ±12 | 35 ±8 |
| **RER** | 0.84 ±0.06 | 0.84 ±0.10 | | 0.85 ±0.09 | 0.78 ±0.07 | 0.79 ±0.15 | 0.75 ±0.07 | 0.76 ±0.06 | 0.78 ±0.09 | 0.79 ±0.08 | 0.75 ±0.06 | 0.77 ±0.06 | 0.71 ±0.05 |
| **CHO ox (% E)** | 44 ±20 | 47 ±32 | | 50 ±30 | 29 ±24 | 20 ±27 | 19 ±19 | 21 ±17 | 29 ±27 | 29 ±24 | 17 ±14 | 22 ±19 | 9 ±12 |
| **Fat ox (% E)** | 56 ±20 | 53 ±32 | | 50 ±30 | 71 ±24 | 60 ±27 | 81 ±19 | 79 ±17 | 71 ±27 | 71 ±24 | 83 ±14 | 78 ±19 | 91 ±12 |
| ***During exercise*** | | | | | | | | | | | | | |
| **EE (kcal·min^-1^)** | 10.73±2.13 | 10.91±1.54 | | 10.47±2.4 | 10.88±1.45 | 10.52±1.7 | 11.28±2.26 | 15.25±2.27 | 14.21±1.77 | 14.99±2.56 | 14.62±2.58 | 14.40±2.76 | 14.46±2.83 |
| **Fat ox (kcal·min^-1^)** | 3.54 ±1.86 | 3.21 ±2.50 | | 3.58 ±2.51 | 4.19 ±2.82 | 3.54 ±2.84 | 4.70 ±3.13 | 8.5 ±4.0 | 7.2 ±3.9 | 7.3 ±3.7 | 7.8 ±3.8 | 8.4 ±2.9 | 9.3 ±4.1 |
| **CHO ox (kcal·min^-1^)** | 7.19 ±2.05 | 7.70 ±3.05 | | 6.89 ±2.28 | 6.69 ±2.59 | 7.01 ±2.92 | 6.58 ±2.98 | 6.8 ±3.5 | 7.0 ±4.2 | 7.7 ±3.2 | 6.8 ±3.6 | 6.0 ±2.7 | 5.2 ±3.4 |
| **Fat ox (% E)** | 32 ±16 | 30 ±26 | | 34 ±23 | 38 ±26 | 33 ±25 | 40 ±27 | 55 ±22 | 51 ±28 | 49 ±21 | 53 ±22 | 58 ±17 | 63 ±25 |
| **CHO ox (% E)** | 68 ±16 | 70 ±26 | | 66 ±23 | 62 ±26 | 67 ±25 | 60 ±27 | 45 ±22 | 49 ±28 | 51 ±21 | 47 ±22 | 42 ±17 | 37 ±25 |
| **RER** | 0.91 ±0.05 | 0.92 ±0.08 | | 0.90 ±0.08 | 0.88 ±0.08 | 0.91 ±0.08 | 0.90 ±0.07 | 0.84 ±0.07 | 0.86 ±0.10 | 0.85 ±0.07 | 0.85 ±0.07 | 0.83 ±0.05 | 0.82 ±0.08 |

Data are presented as mean ± standard deviation; % E: % of energy expenditure; CHO ox: Carbohydrate oxidation.
